# Family‐centred care in cystic fibrosis: a pilot study in North Queensland, Australia

**DOI:** 10.1002/nop2.84

**Published:** 2017-05-11

**Authors:** Wendy Smyth, Gail Abernethy, Melanie Jessup, Tonia Douglas, Linda Shields, AREST‐CF

**Affiliations:** ^1^ Nursing Research Unit, Townsville Hospital and Health ServiceTownsvilleQueenslandAustralia; ^2^ College of Healthcare SciencesJames Cook UniversityTownsvilleQueenslandAustralia; ^3^ Tropical Health Research UnitTownsville Hospital and Health ServiceTownsvilleAustralia; ^4^ Australian Catholic University/Metro North Hospital and Health ServiceBrisbaneQueenslandAustralia; ^5^ Queensland Children's Medical Research InstituteThe University of QueenslandBrisbaneQueenslandAustralia; ^6^ Respiratory Unit, Lady Cilento Children's HospitalSouth BrisbaneQueenslandAustralia; ^7^ Curtin UniversityBentleyWestern AustraliaAustralia; ^8^ Faculty of Science, Charles Sturt UniversityBathurstNew South WalesAustralia; ^9^ School of MedicineThe University of QueenslandBrisbaneQueenslandAustralia

**Keywords:** cystic fibrosis, family‐centred care, nurses, nursing, rural and remote health care

## Abstract

**Aims:**

The aims were to: (i) examine perceptions of family‐centred care of parents of children with cystic fibrosis and healthcare professionals who care for them; (ii) test design and tools in a regional population.

**Design:**

Quantitative pilot study of existing questionnaire.

**Methods:**

The methods involved were comparative, cross‐sectional survey of parents of children with cystic fibrosis and health staff in North Queensland, using “Perceptions of Family Centered Care – Parent” and “Perceptions of Family Centered Care – Staff” questionnaires; and descriptive study of tools.

**Results:**

Eighteen staff, 14 parents (78%, 61%); using Mann–Whitney U, showed no significant differences in scores in categories: ‘support’ ‘respect’, ‘collaboration’. Comments about suitability of questionnaires varied, but were largely positive.

## 1 Introduction

Family‐centred care (FCC) is endorsed and accepted as the standard of paediatric health care delivery across paediatric clinical institutions in Australia and is recognized as a dimension of healthcare quality in its own right (Australian Commission on Safety and Quality in Healthcare, 2011). Broadly, FCC is defined as providing care in partnership with families and children: “a way of caring for children and their families in health services which ensures that care is planned around the whole family, not just the individual child/person and where all the family members are recognized as care recipients” (Shields, Pratt, & Hunter, 2006, p. 1318).

Family‐centred care is based on a set of core principles: dignity and respect; information sharing; participation and collaboration; negotiation and care in context (Institute for Patient and Family‐Centered Care, 2010). These principles aim to improve quality and safety of paediatric health care through enhanced patient/family experience and communication and translation into improved health outcomes for children. Evidence for better health outcomes through FCC is relatively limited (Shields et al., 2012), although studies demonstrate improved child psychological health, satisfaction, family functioning, improved access to healthcare, reduced emergency presentations and better health service efficiency (Mikkelsen & Frederiksen, 2011; Smith, Swallow, & Coyne, 2015). Family‐centred care can improve information gathering and clinical acuity for healthcare professionals (Kuhlthau, Bloom, & Van Celave, 2011).

While FCC is endorsed widely, it is often poorly implemented and misunderstood (Mikkelsen & Frederiksen, 2011; Shields, 2010; Smith et al., 2015). Health professionals may not know how to apply the principles of FCC to everyday practice and patients and families may not appreciate what it means to participate in healthcare decisions, or what they should expect. Perceptions of FCC delivery and implementation may differ between parents and health care professionals and misalignment in expectations or purpose may prevent successful implementation of FCC, for example, problems in communicating information to parents may be exacerbated if the parents recognize that they know more about CF than the health professional does, although the health professional who is trying to communicate something may not understand or accept this (Shields 2015).

Cystic fibrosis (CF) is an inherited multi‐systemic disease, principally affecting the lungs and digestive system. It occurs in approximately 1:2,500 live births (Australian Cystic Fibrosis Registry, 2013) and is life‐limiting (MacKenzie, Gifford, & Sabadosa, 2014). The intensive, daily, lifelong management of CF imposes heavy demands on families and children: the need for frequent hospital visits for ambulatory and inpatient care; navigating health care provided by a large multidisciplinary team; and increasing care complexity creating challenging decisions and interactions (Jessup & Parkinson, 2010). Families with children who have CF and who reside in regional, rural or remote locations have additional burdens related to access to services, travelling time and social isolation. The tenets of centrality and importance of the family in child healthcare planning, make FCC an ideal model for a chronic paediatric condition such as CF (Kuhlthau et al., 2011). While studies have compared perceptions of FCC between parents and health professionals in the paediatric setting (Gill, Pascoe, Monterosso, Young, Burr, Tanner & Shields 2013), there have been no studies examining perceptions of FCC held by parents of children with CF in Australia or elsewhere.

The aim of this study was to compare perceptions of FCC delivery held by parents of children with CF living in regional North Queensland and healthcare professionals who care for them. A secondary aim was to pilot test the study design, preparatory to broadening it to a larger study in this population.

### 1.1 Research questions

- In CF care, what are the parents’ perceptions of family‐centred care?
- In CF care, what are the staff's (nurses, doctors and allied health professionals) perceptions of family‐centred care?
- Do the Perceptions of Family Centered Care – Parent’ (PFCC‐P)’ and the ‘Perceptions of Family Centered Care – Staff (PFCC‐S)’ (Gill et al., 2013; Shields & Tanner, 2004) questionnaires work in rural and remote populations?

## 2 Materials and methods

### 2.1 Design

Pilot of a comparative, cross‐sectional survey of parents of children with CF and staff caring for them, in North Queensland, Australia.

### 2.2 Tools

The Perceptions of Family Centered Care – Parent (PFCC‐P) and the Perceptions of Family Centered Care – Staff (PFCC‐S) questionnaires (Gill et al., 2013; Shields & Tanner, 2004) comprise a set of questionnaires for parents and health professionals that test perceptions held about how the principles of FCC are enacted in practice. These questionnaires were developed and trialled in studies in Australia (Shields & Tanner, 2004) and the UK (Aggarwal et al., 2009). They are essentially the same, with questions matched to examine differences in responses about the delivery of FCC between parents and health professionals. The 20 items comprise three sub‐scales: Respect (six items), Collaboration (nine items) and Support (five items), which in the original questionnaire, were based on items investigated by Galvin et al. (2000). Responses on the PFCC‐P and PFCC‐S were coded as never = 1, sometimes = 2, usually = 3 and always = 4. Gill et al. (2013) (*n* = parents 309, staff 519) found overall scores: parents mean 3.45 (*SD* 0.39, median 3.55 (*SD* 3.25–3.7), staff mean 3.14 (*SD* 0.32), staff median (2.95–3.4), (*p* < 0.001). The questionnaires showed internal consistency and content validity; each questionnaire demonstrated a Cronbach's alpha of greater than 0.7 (Gill et al., 2013). Minimal demographic information was also collected.

For this pilot study, in addition to the PFCC‐P and the PFCC‐S, participants were asked complete a second questionnaire to elicit any difficulties they encountered in completing the main questionnaire and to identify questions that were unclear. Both questionnaires took about 15 min to complete.

### 2.3 Setting

North Queensland, Australia, in 2016.

### 2.4 Sample

The staff sample was purposively selected from the medical, nursing and allied health staff working in the paediatric department of a tertiary‐level hospital, while parents were recruited from the population of parents of children with CF known to the local CF charity and support group.

### 2.5 Recruitment

We distributed packs to nurses, doctors and allied health staff working in the paediatric department of a regional hospital (where children with CF are managed on an ongoing basis). Each pack included the staff participant information sheet, the two questionnaires for staff participants and an internal mail envelope addressed to the research department for return of completed questionnaires. These were distributed by a senior nurse in the paediatric department.

Recruitment of parent participants was conducted via the local CF charity organization with emphasis on the voluntary and anonymous nature of the study. Participants were provided with a study pack, which included the parent participant information sheet, the two questionnaires for parent participants and a postage‐paid envelope addressed to the nursing research unit for return of completed questionnaires. The required number of study packs for parents were assembled by the research team and distributed to parents of children with CF in the region through the local charity coordinator. In this way, the respondents remained anonymous to the researchers.

### 2.6 Analysis

Data were entered into SPSS v 22 for analysis. Demographic information related to participants are reported as frequencies and percentages. A mean score was calculated for each of the three sub‐scales: respect, collaboration and support. Negatively worded items were reverse‐coded prior to calculation. Because the data were not normally distributed, we used non‐parametric Mann–Whitney U tests to examine differences between the groups.

### 2.7 Ethics

Ethics approval was given by the Hospital and Health Service's Human Research Ethics Committee (HREC/13/QTHS/189) and acknowledged by James Cook University Human Research Ethics Committee (H5406). Return of the anonymous questionnaires implied consent.

## 3 Results

Eighteen staff and 14 parents returned completed questionnaires (response rate of 78% and 61% respectively).

The staff group was younger than the parent group, with 11 (61%) members younger than 31 years, whereas all the parents were 31 years or older (Tables 1 and 2). Both groups of participants were well educated. The length of experience working with children among staff participants ranged from 6 months ‐ 37 years. Most parents lived relatively close to the hospital, with only three (21%) living more than an hour's travel time from the hospital. One parent had two children with CF; the remaining 13 parents had one child with CF.

**Table 1:** Demographics of parent participants

Parent (*N *= 14)	*N*	%
Age		
31–45 years	12	85.7
46–50 years	1	7.1
Not specified	1	7.1
Highest level of education		
High school certificate	4	28.6
TAFE	4	28.6
Undergraduate degree	5	35.7
Postgraduate qualification	1	7.1
Time to travel to hospital		
Less than 30 min	10	71.4
30–60 min	1	7.1
1–4 hr	3	21.4
Difficulty in attending the hospital		
Not much	6	20.0
Little bit	4	28.6
Fair bit	3	21.4
Very difficult	1	7.1
Another person to help		
Yes	11	78.6
No	3	21.4

**Table 2:** Demographics of staff participants

Staff (*N* = 18)	*n*	%
Age		
Less than or equal to 25 years	3	16.7
26–30 years	8	44.4
31–45 years	3	16.7
46–50 years	1	5.6
51–55 years	1	5.6
Over 55 years	2	11.1
Highest level of education		
Undergraduate degree	11	64.7
Postgraduate qualification	5	29.4
Not specified	2	11.1
Occupation		
Nurse	13	72.2
Doctor	3	16.7
Allied health professional	2	11.1
Specialist paediatric qualification		
Yes	9	50.0
No	9	50.0

**Figure 1:**
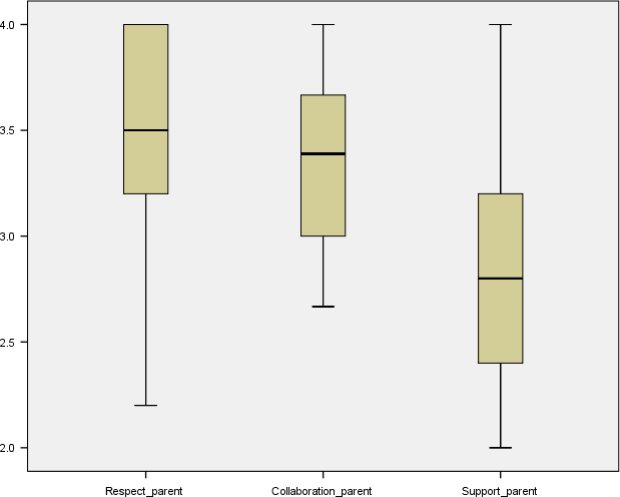
Boxplots (means and interquartile range), parents’ responses

**Figure 2:**
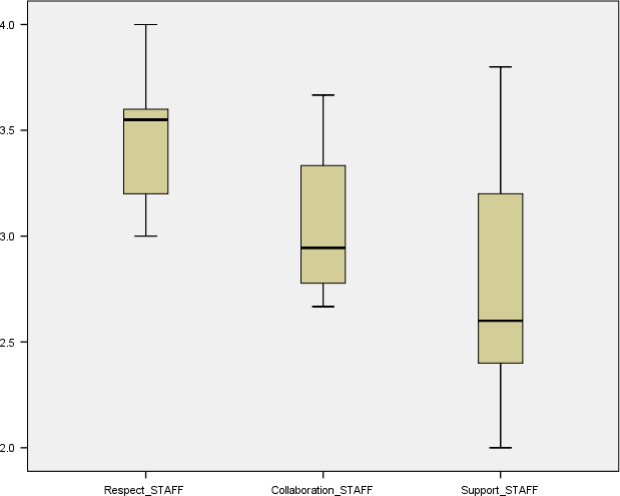
Boxplots (means and interquartile range), staffs’ responses

The maximum score for responses related to perceptions of FCC was ‘4’. The lowest average (median) score for both groups was on the ‘support’ sub‐scale; the scores from both groups for the ‘respect’ and ‘collaboration’ sub‐scales were high (Table 3; Figures 1 & 2). Using the Mann–Whitney U test, no statistically significant differences between parent and staff participant groups on these sub‐scales were found. Both groups were generally positive in their perceptions of FCC in the study setting, with the overall median score being 3.2289 (parents) and 3.025 (staff).

**Table 3:** Parent and staff perceptions of family‐centred care, by sub‐scale and overall

Sub‐scale	Parent median (IQR)	Staff median (IQR)
Respect	3.5000 (3.15–4.00)	3.5500 (3.20–3.60)
Collaboration	3.3889 (2.97–3.72)	2.9444 (2.78–3.33)
Support	2.8000 (2.35–3.25)	2.6000 (2.40–3.25)
Overall	3.2289 (2.78–3.69)	3.0250 (2.86–3.23)

No participant in either group identified any question that did not make sense, or any question that was similar to another. The feedback about the questionnaire from parents was consistently positive, as reflected in comments such as: *‘I think this is an excellent way to gauge how CF children are being care for’*. There were also comments that all the important issues were covered and that the format of the questionnaire allowed for easy completion. However, the staff group were more divided as exemplified by these two general feedback comments: *‘Very broad’* and *‘Clear and concise’*. The parents suggested other questions that could have been asked: the PFCC‐P questionnaire asked about the number of hospital admissions in the preceding year; the suggestion was to ask about visits to hospital, because hospital admissions do not in themselves reflect the amount of healthcare and the contacts with the health professionals.

## 4 Discussion

This study examined the perceptions about the implementation of the principles of FCC held by parents of and health professionals caring for children with CF. Based on their responses to the PFCC‐S and PFCC‐P, both groups were generally positive in their perceptions of FCC in the study setting, with no significant differences between the overall median scores. Thus, the principles of FCC were ‘usually’ implemented.

This is a positive finding, given that the setting was a paediatric service in a generalist regional hospital. However, these median scores were lower than in another Australian study (Gill et al., 2013) and may have been inlfuenced by the small sample size. Compared with Gill et al. (2013), our sample was tiny and we can say little more than that the median scores were different.

The finding that the participants’ average score for the ‘support’ sub‐scale was lower than that for the ‘respect’ and ‘collaboration’ sub‐scales is similar to that in a larger study conducted at two Australian tertiary paediatric hospitals (Gill et al., 2013). The support sub‐scale includes statements such as: ‘The staff are familiar with my child's individual needs’ and ‘The staff understand what my family and I are going through’. Corresponding items on the staff version of the questionnaire are: ‘Staff are familiar with the child's individual needs’ and ‘Staff understand what the parents and their family are going through’. However, unlike that previous study, our study in a smaller regional facility, relating to one specific chronic condition, found no significant difference overall or in sub‐scale scores between parents and health professionals. Because of the differences in the settings and size of samples, the studies therefore are not directly comparable. One other difference between these Australian studies is that the age of the staff respondents was considerably older in the previous study (Gill et al., 2013). However, the small sample size of this pilot is possibly affecting such outcomes and the planned larger study may yield different results.

### 4.1 Limitations

This pilot study used a very small, purposive population with the aim of testing whether or not the questionnaires would work in this particular group of parents of children with a rare but burdensome disease—CF—and the staff who cared for these families. Perhaps the request to complete a set of questionnaires was seen as an added burden to their already busy lives caring for their child with CF (Jessup & Parkinson, 2010) and we strove to distance ourselves from the parents by distributing the study packs via the CF charity. This helped to assure respondents of their anonymity. Children were not included. The uniformity of scores between the participant groups may also have been a reflection of the small sample size. We are limited as to the detail we can publish about the demographic characteristics of the families and the setting, because North Queensland is remote with a small, widely dispersed population. Added to this is the rarity of CF in any population. Anonymity was guaranteed and ethically we were very conscious of ensuring we created no further burden for families and staff.

### 4.2 Implications for nursing practice and further research

The results here will be translated into a larger, Australia‐wide study. If perceptions of FCC are different between parents and staff, then these differences may well colour communication between them, potentially to the detriment of the care and clinical outcomes of the child with CF. Such discrepancies have been found to influence care (Gill et al., 2013; Smith et al., 2015; Mitchell, Burmeister, & Chaboyer, 2012) and nursing practice for the care of children with CF and their families will be enhanced if any possible communication breakdown is prevented by being able to tailor care to the needs and perceptions of the families and children.

## 5 Conclusion

Perceptions of FCC held by health professionals and parents were measured and compared in a small pilot study in North Queensland. Results demonstrate the applicability of the questionnaires to this specific group of people and condition. Future uses of the questionnaire could incorporate minor wording amendments suggested by the participants. What we learnt from this study in one regional area of Australia will be incorporated into an Australia‐wide study about children and families living with CF, to produce evidence to guide development of care plans and communication strategies and to educate and support health professionals and parents. Ultimately, the evidence will be able to be translated into practice and the consequent improvements in care delivery on application of the findings will mean improved clinical outcomes for the children alongside psychosocial outcomes for the families and community.

## Conflicts of interest

No conflict of interest has been declared by the authors.

## Author contributions

All authors contributed to writing and editing the paper. Wendy Smyth managed the project, organized data collection and ethics approval. Gail Abernethy, Melanie Jessup and Wendy Smyth analysed and collected data. Tonia Douglas and Linda Shields conceptualized and initiated the project through AREST‐CF: https://arestcf.telethonkids.org.au/.
